# Draft Genome Sequence of *Cercospora* cf. *sigesbeckiae*, a Causal Agent of Cercospora Leaf Blight on Soybean

**DOI:** 10.1128/genomeA.00708-17

**Published:** 2017-09-07

**Authors:** Sebastian Albu, Sandeep Sharma, Burton H. Bluhm, Paul P. Price, Raymond W. Schneider, Vinson P. Doyle

**Affiliations:** a Department of Plant Pathology and Crop Physiology, Louisiana State University Agricultural Center, Baton Rouge, Louisiana, USA; b Department of Plant Pathology, University of Arkansas, Division of Agriculture, Fayetteville, Arkansas, USA; c Louisiana State University Agricultural Center, Macon Ridge Research Station, Winnsboro, Louisiana, USA

## Abstract

*Cercospora* cf.* sigesbeckiae* is an ascomycete fungal pathogen that infects various plants, including important agricultural commodities, such as soybean. Here, we report the first draft genome sequence and assembly of this pathogen.

## GENOME ANNOUNCEMENT

*Cercospora* cf. *sigesbeckiae* is a broad generalist pathogen infecting at least eight different plant families and causing Cercospora leaf blight (CLB) on soybean (1). Until recently, *Cercospora kikuchii* was thought to be the only causal agent of CLB, but phylogenetic analyses of cercosporoid fungi isolated from infected soybean revealed the presence of two other morphologically similar *Cercospora* species, including *Cercospora* cf.* sigesbeckiae* from leaves and *Cercospora* cf. *flagellaris* from seeds and leaves (1, 2). Prior to 2015, most records of *Cercospora* cf. *sigesbeckiae* came from Asia (3), but recent studies also reported this pathogen from Argentina (2) and Louisiana, USA (1).

CLB infections are characterized by leaf bronzing and typically coincide with seed set. Abaxial foliar lesions are initially observed within the canopy and later develop on lower leaves, stems, and petioles. CLB-associated yield losses have occurred more frequently in the Gulf South since 1999 (4), and losses have also been reported from other soybean-growing regions in the United States (5, 6) and South America (7, 8). Management strategies to control CLB have traditionally relied on a quinone outside inhibitor, methyl benzimidazole carbamate, and thiophanate methyl foliar fungicides. However, repeated application of these compounds has resulted in the development of resistant isolates (9, 10).

More than 650 species of *Cercospora* have been described (11), although only 4 published genomes (of *C. arachidicola*, *C. zeae-maydis*, *Cercospora* aff. *canescens*, and *C. sojina*) exist, with only *C. sojina* being an important soybean pathogen. There are also no molecular barcodes available to reliably discriminate among closely related *Cercospora* species. Therefore, additional genomic data may facilitate the development of species-specific markers for accurate identification, which could, in turn, add to an integrated defense management approach for CLB through improved genetic resistance in soybean cultivars.

*Cercospora* cf.* sigesbeckiae* strain PP_2012_071 was isolated from foliar lesions on soybean collected in Louisiana. Genomic DNA was isolated from hyphal tissue using a modified cetyltrimethylammonium bromide (CTAB) extraction protocol developed in our laboratory and was quantified using a Qubit fluorometer (Invitrogen, Carlsbad, CA, USA). Libraries were constructed using a NEBNext Fast DNA fragmentation & library prep set for Ion Torrent (New England BioLabs, Inc., Ipswich, MA, USA), evaluated for quality and size using the Agilent 2200 TapeStation system (Agilent Technologies, Santa Clara, CA, USA), and sequenced on an Ion Personal Genome Machine system using an Ion 318 Chip version 2. A total of 1.63 Gbp and 6,050,000 sequence reads were obtained, with a median read length of 281 bp. A total of 5,770,664 reads were assembled *de novo* using MIRA 4.0.2 (12), resulting in 469 contigs of at least 500 bp, with a total consensus length of 34,132,478 bp, largest contig size of 1,591,857 bp, and *N*_50_ value of 418,495 bp. The maximum total coverage of the assembly was 2,042×, with an average total coverage of 43.06× calculated from contigs of at least 5,000 bp and 13× coverage, with a G+C content of 51.5%.

### Accession number(s).

This whole-genome shotgun project has been deposited at DDBJ/ENA/GenBank under the accession no. NKQR00000000. The version described in this paper is version NKQR01000000.

## References

[1] Albu S, Schneider RW, Price PP, Doyle VP 2016 *Cercospora* cf. *flagellaris* and *Cercospora* cf. *sigesbeckiae* are associated with *Cercospora* leaf blight and purple seed stain on soybean in North America. Phytopathology 106:1376–1385. doi:10.1094/PHYTO-12-15-0332-R. 27183302

[2] Soares AP, Guillin EA, Borges LL, Silva AC, Almeida ÁM, Grijalba PE, Gottlieb AM, Bluhm BH, Oliveira LO 2015 More *Cercospora* species infect soybeans across the Americas than meets the eye. PLoS One 10:e0133495. doi:10.1371/journal.pone.0133495. 26252018 PMC4529236

[3] Groenewald JZ, Nakashima C, Nishikawa J, Shin HD, Park JH, Jama AN, Groenewald M, Braun U, Crous PW 2013 Species concepts in *Cercospora*: spotting the weeds among the roses. Stud Mycol 75:115–170. doi:10.3114/sim0012. 24014899 PMC3713887

[4] Cai G, Schneider RW, Padgett GB 2009 Assessment of lineages of *Cercospora kikuchii* in Louisiana for aggressiveness and screening soybean cultivars for resistance to *Cercospora* leaf blight. Plant Dis 93:868–874. doi:10.1094/PDIS-93-9-0868. 30754540

[5] Hershman D 2009 *Cercospora* leaf blight in Kentucky. Plant pathology fact sheet PPFS-AG-S-20. University of Kentucky Cooperative Extension Service, Lexington, KY http://plantpathology.ca.uky.edu/files/ppfs-ag-s-20.pdf.

[6] Geisler LG 2013 Purple seed stain and *Cercospora* blight. University of Nebraska, Lincoln, NE http://cropwatch.unl.edu/plantdisease/soybean/purple-seed-stain.

[7] Almeida ÁMR, Piuga FF, Marin SRR, Binneck E, Sartori F, Costamilan LM, Teixeira MRO, Lopes M 2005 Pathogenicity, molecular characterization, and cercosporin content of Brazilian isolates of *Cercospora kikuchii*. Fitopatol Bras 30:594–602. doi:10.1590/S0100-41582005000600005.

[8] Wrather A, Shannon G, Balardin R, Carregal L, Escobar R, Gupta GK, Ma Z, Morel W, Ploper D, Tenuta A 2010 Effect of diseases on soybean yield in the top eight producing countries in 2006. Plant Health Prog doi:10.1094/PHP-2010-0125-01-RS.

[9] Price PP, Purvis MA, Cai G, Padgett GB, Robertson CL, Schneider RW, Albu S 2015 Fungicide resistance in *Cercospora kikuchii*, a soybean pathogen. Plant Dis 99:1596–1603. doi:10.1094/PDIS-07-14-0782-RE. 30695960

[10] Albu S, Price PP, Doyle VP, Padgett B, Schneider RW 2016 The G143A mutation is responsible for strobilurin fungicide resistance in *Cercospora* leaf blight and purple seed stain pathogens of Louisiana soybean. Plant Health Prog 17:197.

[11] Crous PW, Braun U 2003 *Mycosphaerella* and its anamorphs: 1. Names published in *Cercospora* and *Passalora*. CBS Biodiversity Series 1. National History Book Service, Utrecht, the Netherlands.

[12] Chevreux B, Wetter T, Suhai S 1999 Genome sequence assembly using trace signals and additional sequence information, p 45–56. *In* Computer science and biology. Proceedings of the German Conference on Bioinformatics, GCB ’99. GCB, Hannover, Germany.

